# Directed Self-Assembly of an Acid-Responsive Block Copolymer for Hole-Shrink Process and Pattern Transfer

**DOI:** 10.3390/nano15201571

**Published:** 2025-10-16

**Authors:** Jianghao Zhan, Jiacheng Luo, Zixin Zhuo, Caiwei Shang, Zili Li, Shisheng Xiong

**Affiliations:** 1 Center of Micro-Nano System, School of Information Science and Technology, Fudan University, Shanghai 200438, China; 21110720052@m.fudan.edu.cn (J.Z.);; 2 Zhangjiang Laboratory, 100 Haike Road, Shanghai 201204, China

**Keywords:** block copolymers (BCPs), polystyrene-block-poly (methyl methacrylate) (PS-*b*-PMMA), Schiff base bond, acid-responsive, thin film self-assembly, wet etching, directed self-assembly (DSA), pattern transfer

## Abstract

Directed self-assembly (DSA) of polystyrene-block-poly (methyl methacrylate) (PS-*b*-PMMA) has garnered substantial interest for semiconductor manufacturing, particularly for fabricating contact holes and vias. However, its application is limited by the low etch selectivity between the PS and PMMA domains. Here, we report an acid-responsive block copolymer, PS-N=CH-PMMA, incorporating a Schiff base (-N=CH-) linkage between the two blocks to impart acid sensitivity. The copolymer is synthesized via aldehyde-terminated PMMA (PMMA-CHO) precursors and is fully compatible with conventional thermal annealing workflows used for PS-*b*-PMMA. Uniform thin films with vertically oriented cylindrical domains were obtained, which could be directly converted into high-fidelity PS masks through acetic acid immersion without UV exposure. Graphoepitaxial DSA in 193i pre-patterned templates produced shrink-hole patterns with reduced critical dimension (CD) and improved local CD uniformity (LCDU). The shrink-hole CD was tunable by varying PMMA-CHO molecular weights. XPS confirmed selective cleavage of Schiff base linkages at the PS/PMMA interface under acidic conditions, while Ohta–Kawasaki simulations indicated interfacial wetting asymmetry governs etch fidelity and residual layer formation. Pattern transfer into TEOS layers was achieved with minimal CD loss. Overall, the acid-cleavable BCP enables scalable, high-fidelity nanopatterning with improved etch contrast, tunable process windows, and seamless integration into existing PS-*b*-PMMA lithography platforms.

## 1. Introduction

As semiconductor devices continue to scale down, there is a growing demand in integrated circuit fabrication for high-resolution nanoscale patterning technologies [1,2]. Conventional lithographic techniques, particularly deep ultraviolet (DUV) lithography, are fundamentally constrained by the diffraction limits of optical systems, making it challenging to meet the stringent requirements for pattern resolution and complexity [3]. Although extreme ultraviolet (EUV) lithography alleviates some limitations due to its shorter wavelength, it still faces significant challenges, including stochastic defects and high system costs [4,5]. Although unconventional patterning techniques such as micromoulding in capillarity and lithographic-controlled wetting can direct patterning of semiconductors to generate ordered micro/nanostructures, their resolution remains insufficient for producing high-fidelity nanopatterns required for advanced semiconductor applications [6,7]. Directed self-assembly (DSA) of block copolymers (BCPs) is a promising complementary lithography technique to extend the resolution limits of conventional photolithography for industrial manufacturing [8,9,10]. Leveraging microphase separation, BCPs can self-assemble into various nanostructures, such as lamellae, cylinders, or spheres, by precisely tuning their molecular weight and the volume fraction of each block [11,12]. Moreover, DSA enables the formation of highly ordered, large-area periodic patterns when guided by physical or chemical templates [13,14,15]. This technique offers high-resolution patterning at a lower cost while remaining compatible with existing semiconductor manufacturing processes [16].

In semiconductor logic chip fabrication, contact holes connect the top metal layer to the active regions of transistors, whereas vias serve as vertical interconnects between successive metal layers [17,18]. Both structures are critical for maintaining electrical continuity in multilayer interconnect architectures. As feature sizes continue to shrink, the precise fabrication of nanoscale structures with small critical dimensions (CDs) becomes increasingly complex using conventional photolithography [19]. Graphoepitaxial DSA of BCPs within pre-patterned confinement templates presents a viable strategy for generating high-resolution, high-fidelity contact holes and vias, which are essential for improving device performance, reducing power consumption, and enhancing overall system reliability [20]. Among various BCPs, polystyrene-block-polymethyl methacrylate (PS-*b*-PMMA) is widely employed in DSA due to its excellent self-assembly capabilities [21]. A key step in the DSA process involves selectively removing the PMMA phase after assembly to form the desired patterns. However, existing etching methods for PS-b-PMMA, including dry and wet etching, have significant limitations [22]. Dry etching processes, such as reactive ion etching (RIE) and inductively coupled plasma (ICP) etching, utilize gas plasmas to remove polymer domains selectively. Various gas-plasma chemistries have been optimized to enhance etch selectivity between PS and PMMA. Representative examples include oxygen/argon (O_2_/Ar) [23], methane/oxygen (CH_4_/O_2_) [24], methane/nitrogen (CH_4_/N_2_) [25], carbon tetrafluoride/oxygen (CF_4_/O_2_) [26], octafluorocyclobutane/oxygen(C_4_F_8_/O_2_) [27], trifluoromethane/oxygen (CHF_3_/O_2_) [28], carbon monoxide/argon (CO/Ar) [22], and carbon monoxide/hydrogen (CO/H_2_) [29]. Among these, plasma etching with O_2_/Ar is extensively employed due to its ability to produce smooth PS mask surfaces and its broad compatibility with different substrates [30,31]. Despite these advantages, the etch selectivity between PS and PMMA in O_2_/Ar plasma remains relatively low, typically ranging from 2:1 to 4:1 [32]. This limited selectivity leads to undesired vertical loss of the PS mask and lateral etching due to the isotropic nature of the plasma, thereby distorting feature dimensions and compromising pattern fidelity and resolution [33,34], especially for hole patterns. To address this limitation, wet etching strategies have been proposed based on the differential solubility of the two blocks in acid solutions [35]. Under UV exposure, PMMA chains undergo photo-degradation, breaking into low-molecular-weight, readily dissolved fragments, while PS domains crosslink, reducing their solubility in organic solvents [36]. This contrast enables the selective removal of PMMA using solvents such as acetic acid while preserving the integrity of the PS matrix [37]. However, UV pretreatment can lead to non-uniform photochemical reactions, affecting pattern fidelity [38]. In addition, the covalent linkage between the PS and PMMA blocks often leaves residual PMMA at the interface, resulting in incomplete removal, pore shrinkage, and a narrow process window [39]. Although increasing the exposure dose and duration can facilitate the removal of PMMA residues, it frequently leads to excessive crosslinking of the PS phase, causing pattern shrinkage and a deterioration in pattern fidelity [33]. Introducing an acid-responsive junction into the backbone of BCPs provides an effective strategy for controlling the cleavage between blocks, thereby addressing the limitations above [40]. Schiff base bonds, a class of dynamic covalent bonds, have garnered considerable interest in the design of acid-responsive materials due to their high sensitivity to pH variations [41]. These imine linkages, formed via the condensation of aldehyde or ketone groups with amines, are readily cleaved under acidic conditions and can be reformed in alkaline environments [42]. Schiff bases derived from benzaldehyde can form stable imine bonds under mild conditions, with minimal sensitivity to water accumulation during condensation [43,44]. Significant progress has been made in developing dynamic BCPs featuring Schiff base linkages, including polystyrene-r-poly (ethylene oxide) (PS-r-PEO) for nanoporous materials [45] and polystyrene-r-poly (ethylene glycol) (PS-r-PEG) for drug delivery applications [46], where “r” denotes a reversible covalent bond. In our previous study, an acid-cleavable lamellar PS-r-PMMA was developed for direct wet development and high-fidelity line/space patterning [47]. Building upon this concept, the present work expands from lamellae to cylinders, and systematically explores the underlying self-assembly mechanism.

**Scheme 1:**
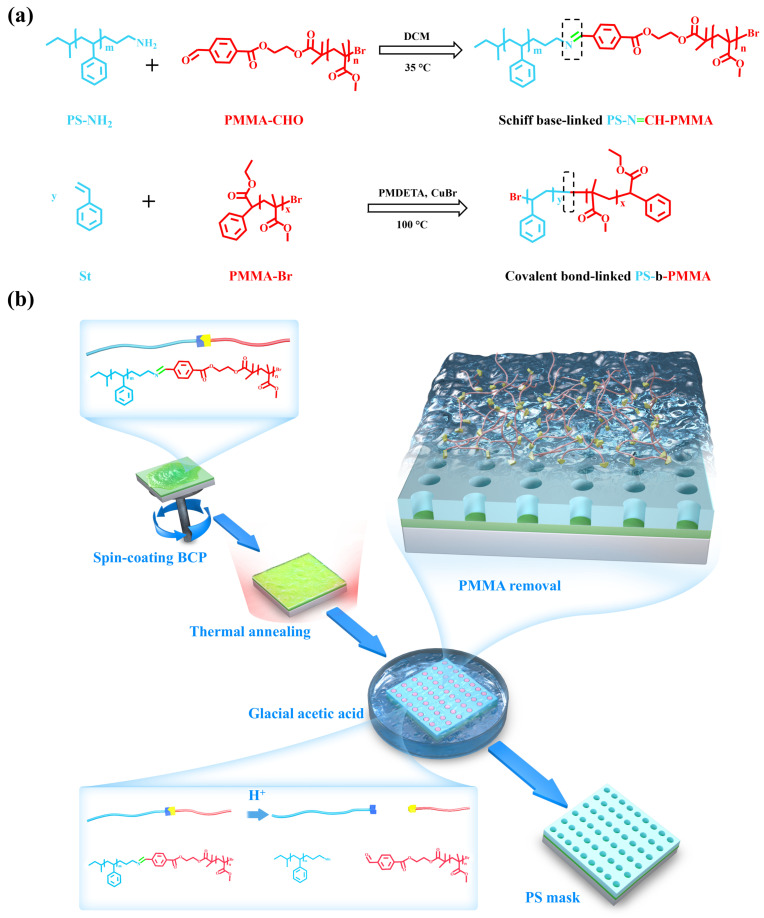
The schematic design strategy of BCPs, self-assembled thin films, and wet etching processes. (**a**) Synthetic route of functional PS-N=CH-PMMA BCPs and conventional PS-*b*-PMMA BCP. (**b**) Process flow for fabricating a PS mask via wet etching of PS-N=CH-PMMA.

Herein, we report the design and synthesis of a cylindrical-phase Schiff base-linked BCP, PS-N=CH-PMMA, in which an aromatic aldehyde-derived Schiff base bond is incorporated between the PS and PMMA segments (as illustrated in Scheme 1). This copolymer exhibits self-assembly (SA) and directed self-assembly (DSA) behavior under thermal annealing conditions comparable to the conventional PS-*b*-PMMA. Notably, the PMMA domain can be efficiently and selectively removed via mild acetic acid treatment without UV exposure, thereby significantly simplifying the wet etching process while preserving the structural integrity of the PS matrix. X-ray photoelectron spectroscopy (XPS) further elucidated the etching mechanism, which confirmed the effective removal of the PMMA component. Furthermore, DSA shrink-hole experiments demonstrated that the BCP can generate large-area, defect-free shrink-hole patterns following wet etching. In combination with Ohta-Kawasaki theoretical simulations and interfacial wetting analysis, it was revealed that the surface chemical characteristics of the template hole—specifically, PMMA-preferential wetting at the bottom and PS-preferential wetting along the sidewalls—play a critical role in the formation of residual PS layers. Finally, successful shrink-hole pattern transfer from the PS mask into the underlying TEOS layer yielded high pattern fidelity. These findings highlight the potential of PS-N=CH-PMMA as a promising material platform for advancing DSA lithography.

## 2. Materials and Methods

### 2.1. Materials

Amino-terminated polystyrene (PS-NH_2_, M_n_ = 46 kg/mol, PDI = 1.07) and random copolymer hydroxyl-terminated poly(styrene-co-methyl methacrylate) (P20255a, M_n_ = 8 kg/mol, F_St_ = 72%, PDI = 1.17) were purchased from Polymer Source Inc. Ethyl α-bromophenylacetate (EBPA), 2-hydroxyethyl 2-bromo-2-methylpropanoate (HEBIB), 4-dimethylaminopyridine (DMAP), 1-(3-dimethylaminopropyl)-3-ethylcarbodiimide hydrochloride (EDC·HCl), and 4-formylbenzoic acid were obtained from Aladdin and used as received. Cuprous bromide (CuBr), anhydrous diethyl ether, propylene glycol methyl ether acetate, anhydrous methanol, tetrahydrofuran (THF), anisole, N,N,N′,N″,N″-pentamethyldiethylenetriamine (PMDETA), styrene (St), methyl methacrylate (MMA) and glacial acetic acid were purchased from Sinopharm Chemical Reagent Co., Ltd. Silicon wafers were obtained from Suzhou Research Material Micro Nano Technology Co., Ltd. Styrene and methyl methacrylate were passed through neutral alumina columns to remove inhibitors before use. CuBr was purified with a dilute acetic acid solution, followed by acetone, and then dried under a vacuum. All other solvents were used without further purification. The deep hole array guiding template fabricated by 193i lithography was provided by the Shanghai Integrated Circuit Research and Development Center (ICRD).

### 2.2. Synthesis of Bromide-Terminated Poly (Methyl Methacrylate) Homopolymer (PMMA_20k_-Br)

PMMA_20k_-Br was synthesized following the method reported by Chatterjee et al. [48]. Ethyl α-bromophenylacetate (0.243 g, 1.07 mmol), PMDETA (0.173 g, 1.18 mmol), methyl methacrylate (100 mL, 940 mmol), and anisole (200 mL) were successively added to a 500 mL Schlenk bottle. The reaction mixture was deoxygenated with nitrogen for 1 h, and then cuprous bromide was added under a nitrogen atmosphere. The reaction was then conducted in an oil bath at 60 °C for 2 h, followed by quenching with liquid nitrogen. After dilution with tetrahydrofuran, a neutral alumina column was used to remove the copper salt. Finally, the product was precipitated in anhydrous methanol and dried in a vacuum oven for 48 h (denoted PMMA-Br). ^1^H NMR of PMMA_20k_-Br (400 MHz, CDCl_3_, δ): 0.80–1.10 ppm (m, -C(CH_3_)-), 3.60 ppm (s, -OCH_3_).

### 2.3. Synthesis of Conventional Polystyrene-Block-Poly (Methyl Methacrylate) BCP (PS_40k_-b-PMMA_20k_)

PS_40k_-*b*-PMMA_20k_ was synthesized based on the approach described by Huang et al. [49]. PMMA_20k_-Br (10 g, 0.5 mmol) was dissolved in a 250 mL reaction vessel containing PMDETA (0.0865 g, 0.5 mmol) and styrene (52 g, 500 mmol). The solution was deoxygenated with nitrogen for 1 h. Subsequently, CuBr was added, and nitrogen deoxygenation continued for 30 min. The reaction vessel was immersed in an oil bath at 100 °C for 5 h. After the reaction, the mixture was diluted with THF and passed through a neutral alumina chromatographic column to remove the copper salt. The eluate was collected and precipitated in anhydrous methanol to form the white solid. Fractional precipitation was performed using a solvent pair of THF and methanol to remove unreacted PMMA. The remaining white solid was washed with cyclohexane to remove any residual PS. Finally, the product was obtained by vacuum drying at 100 °C for 48 h. ^1^H NMR of PS_40k_-b-PMMA_20k_ (400 MHz, CDCl_3_, δ): 0.80–1.20 ppm (m, -C(CH_3_)- of PMMA), 3.60 ppm (s, -OCH_3_ of PMMA), 6.30–7.30 ppm (m, Ar-H, aromatic protons of PS).

### 2.4. Synthesis of Hydroxyterminated Poly (Methyl Methacrylate) Homopolymers (PMMA-OH)

PMMA-OH synthesis was carried out according to the method described by Tamura et al. [50]. HEBIB (0.211 g, 1 mmol), PMDETA (0.173 g, 1 mmol), MMA (100 mL, 0.9 mol), and anisole (200 mL) were successively added to a 500 mL Schlenk flask. The mixture was deoxygenated with nitrogen for 1 h. CuBr (0.14 g, 1 mmol) was added, and the reaction mixture was further deoxygenated with nitrogen for 30 min. The reaction flask was heated at 60 °C. The polymerization was terminated by rapidly cooling the reaction mixture with liquid nitrogen. The copper salt was removed by passing the diluted reaction mixture through a neutral alumina column using tetrahydrofuran (THF) as the eluent. The eluate was collected, and the polymer was precipitated in cold anhydrous methanol. The white precipitate was collected by filtration, redissolved in THF, and reprecipitated in methanol twice to remove any unreacted monomer and impurities. The resulting white solid was dried under vacuum at 40 °C for 48 h to obtain the PMMA-OH homopolymer. By controlling the polymerization time, PMMA-OH homopolymers with number-average molecular weights (M_n_) of 20, 23, and 26 kg mol^−1^ were sequentially synthesized, denoted PMMA_20k_-OH, PMMA_23k_-OH, and PMMA_26k_-OH, respectively. ^1^H NMR of PMMA-OH (400 MHz, CDCl_3_, δ): 0.80–1.20 (m, -C(CH_3_)-), 3.55 (s, -OCH_3_), 4.10 (t, -OH).

### 2.5. Synthesis of Aromatic Aldehyde-Terminated Poly (Methyl Methacrylate) Homopolymers (PMMA-CHO)

PMMA-CHO polymers were synthesized by an esterification reaction based on the methodology developed in previous research [42]. Taking PMMA_20k_-OH as an example, PMMA_20k_-OH (20.127 g, 1 mmol), DMAP (0.134 g, 0.1 mmol), EDC·HCl (2.301 g, 12 mmol), and 4-formylbenzoic acid (1.634 g, 12 mmol) were added to a 250 mL round-bottom flask. Dichloromethane (100 mL) was then introduced into the flask, and the reaction mixture was stirred at 35 °C for 72 h. On completion, the mixture was precipitated into anhydrous diethyl ether to yield a white solid. The solid obtained was repeatedly washed with a solvent mixture of tetrahydrofuran and anhydrous methanol to remove residual reactants and impurities. Finally, the purified product was dried in a vacuum oven at 40 °C for 48 h. ^1^H NMR of PMMA20k-CHO (400 MHz, CDCl_3_, δ): 0.80–1.20 ppm (m, -C(CH_3_)-), 3.55 ppm (s, -OCH_3_), 7.80–8.20 ppm (m, Ar-H), 10.00 ppm (s, -CHO).

### 2.6. Synthesis of Schiff Base-Linked PS-PMMA BCP (PS-N=CH-PMMA)

PS-N=CH-PMMA BCPs were obtained via a Schiff base reaction [51]. By maintaining a constant molecular weight of PS_46k_-NH_2_ and systematically varying the molecular weight of PMMA-CHO, the three distinct BCPs with different domain spacing (*L*_0_) were synthesized. For example, in a 50 mL round-bottom flask, PMMA_23k_-CHO (0.23 g) and PS_46k_-NH_2_ (0.46 g) were dissolved in 20 mL of dichloromethane. The reaction mixture was stirred at 35 °C for 24 h. Subsequently, the reaction mixture was precipitated into an excess of anhydrous ether. This fractional purification was performed using tetrahydrofuran (THF) and methanol solvent systems. The resulting precipitate was collected and washed with cyclohexane to remove unreacted PS_46k_-NH_2_. The purified BCP was then dried under vacuum at 40 °C for 48 h. ^1^H NMR (400 MHz, CDCl_3_) δ: 0.80–1.20 ppm (m, CCH_3_), 3.55 ppm (s, OCH_3_), 6.30–7.30 ppm (m, Ar-H), 8.20 ppm (s, -N=CH-).

### 2.7. Synthesis of Random Copolymer Mats with Different PS Molar Fractions (F_St_)

Random copolymer mats, specifically poly (styrene-co-methyl methacrylate-co-glycidyl methacrylate) (abbreviated as Poly (St-co-MMA-co-GMA)), were synthesized via conventional free radical solution polymerization, following the method reported by Liu et al. [52]. The synthesis of Mat72 is presented here as a representative example, where “Mat72” refers to the random copolymer mat with a PS molar fraction of 72%, derived from a defined monomer feed ratio and purification procedure. In a 500 mL round-bottom flask, styrene (82.52 mL, 0.72 mol), methyl methacrylate (27.57 mL, 0.26 mol), and glycidyl methacrylate (2.65 mL, 0.02 mol) were dissolved in 200 mL of anisole. The solution was thoroughly degassed by nitrogen purging for 8 h. Subsequently, AIBN (0.5 mol% relative to total monomer content) was added, and the solution was degassed again for 30 min. Polymerization was conducted at 70 °C in an oil bath under magnetic stirring for 8 h. The reaction was terminated by pouring the mixture into liquid nitrogen. The resulting white polymer was precipitated by excess anhydrous methanol and purified by three additional washings using a mixture of tetrahydrofuran and methanol. The final white solid was dried under vacuum at 45 °C for 72 h. The PS molar fraction (F_St_) in the resulting Mat can be precisely tuned by adjusting the monomer feed ratio during synthesis. The copolymer was characterized by ^1^H NMR (400 MHz, CDCl_3_): δ: 0.80–1.20 ppm (m, 3H, α-CH_3_ of PMMA); 1.20–2.30 ppm (m, CH_2_CH of PS and CH_2_C(CH_3_) of PGMA); 2.64–3.23 ppm (m, CH_2_CH(CH_2_)O of PGMA), 3.55 ppm (s, 3H, -OCH_3_ of PMMA), 3.75–3.85 ppm (m, CH_2_CH(CH_2_)O of PGMA); 4.26‒4.38 (m, CH_2_CH(CH_2_)O of PGMA and CH_2_C(CH_3_) of PMMA); 6.30–7.30 ppm (m, 5H, Ar-H).

### 2.8. Self-Assembly of BCPs

Silicon wafers (1 cm × 1 cm) were cleaned by ultrasonication in ethanol, acetone, and isopropyl alcohol for 5 min each. Afterward, the wafers were treated with oxygen plasma to activate the surface. The films of Mat were then prepared by spin coating 0.5 wt% toluene solutions of Mat (Mat63, Mat65, Mat67, and Mat72) and annealed under vacuum at 250 °C for 10 min to facilitate the formation of the modification films. Subsequently, the wafers were ultrasonicated in toluene for 5 min to remove ungrafted mat material. For the deposition of BCPs, thin films of PS-N=CH-PMMA and PS-*b*-PMMA were prepared by spin-coating 2.0 wt% PGMEA solutions of the respective BCPs onto Mat-coated silicon wafers. Subsequently, these films were thermally annealed under vacuum at 230 °C for 30 min to promote self-assembly of the BCPs and the formation of well-ordered nanostructures.

### 2.9. Directed Self-Assembly of BCPs

Contact hole shrinkage experiments were conducted using the functional PS-N=CH-PMMA BCPs and conventional PS_40k_-*b*-PMMA_20k_ BCP on 193i guiding template wafers. Guiding silicon templates were first cleaned by ultrasonication in isopropyl alcohol for 2 min to remove any surface contaminants. Following surface pretreatment, the P20255a random copolymer brush was spin-coated from a 3.0 wt% solution onto the guiding template. The coated wafers were annealed under vacuum at 220 °C for 10 min to promote brush layer grafting. Any ungrafted brush material was removed by rinsing with PGMEA. Subsequently, the wafers were spin-coated with a 1.5 wt% BCP solution, followed by thermal annealing at 230 °C for 30 min under vacuum to facilitate self-assembly of the BCP into the desired pattern.

### 2.10. Wet Etching Process

A standardized wet etching protocol was applied at room temperature to all self-assembled (SA) and directed self-assembled (DSA) BCP samples. The process involved a 5 min immersion in glacial acetic acid, after which the surface of the samples was thoroughly cleaned of any residual acid using a nitrogen gun, which yielded the final etched samples.

### 2.11. BCP Pattern Transfer

The DSA pattern of the PS_46k_-N=CH-PMMA_23k_ was selected for pattern transfer studies. The PMMA block was selectively removed by wet etching, followed by dry etching using ICP with O_2_ plasma for 10 s to remove PS residues and the brush layer (P20255a). Subsequently, CHF_3_ plasma etching was applied for 210 s to etch the underlying TEOS layer. Then, the SOC layer was removed by immersing the sample in a TMAH solution. These etching and removal steps resulted in well-defined shrink-hole patterns.

### 2.12. Ohta-Kawasaki Model Simulation Method

To investigate the microphase separation behavior of PS-N=CH-PMMA BCPs during the DSA hole-shrink process under varying surface affinities, and to guide the design of hole structural parameters, numerical simulations were carried out using the Ohta-Kawasaki model [53]. This model formulates the polymer phase separation dynamics in terms of a free energy functional incorporating both bulk and surface free energies to account for the hole wall-PMMA interaction. The time-dependent Ginzburg–Landau equation is employed to simulate the evolution of the order parameter, with model parameters set to values reported in the literature (τ = 2.8, g = 1, α = 1). The thickness of the PS residual layer and its effect on hole morphology were studied by simulation under different surface affinity conditions.

### 2.13. Characterization

The proton nuclear magnetic resonance (^1^H NMR) spectra were recorded on a Bruker Avance NEO 400 MHz NMR spectrometer (Bruker Corporation, Billerica, MA, USA), employing chloroform-d as the solvent for all samples. Fourier transform infrared spectroscopy (FTIR) was performed using a Thermo Nicolet iS50 spectrometer (Thermo Fisher Scientific, Waltham, MA, USA), collecting spectra in the range of 400–4000 cm^−1^ with a resolution of 4 cm^−1^ and averaging 32 scans per measurement. The molecular weights and polydispersity indices (PDI) of the polymers were determined by gel permeation chromatography (GPC) using an Agilent 1260 system (Agilent Technologies, Santa Clara, CA, USA); tetrahydrofuran (THF) served as the mobile phase at a flow rate of 1 mL min^−1^, and calibration was conducted using polystyrene standards for molecular weight estimation. Differential scanning calorimetry (DSC) analyses were performed on a Netzsch DSC 200 F3 instrument (NETZSCHGerätebau GmbH, Selb, Germany), within a temperature range of 30 to 200 °C under N_2_ flow.; the heating rate was set at 20 °C min^−1^ during the second heating cycle, and the thermal scans were repeated three times to eliminate the influence of thermal history. Thermogravimetric analysis (TGA) was performed using a Mettler SDTA 851e instrument (Mettler-Toledo GmbH, Greifensee, Switzerland), heating the samples from 30 °C to 800 °C under N_2_ atmosphere at 20 °C min^−1^. X-ray photoelectron spectroscopy (XPS) measurements were performed on a Thermo Scientific K-Alpha instrument (Thermo Fisher Scientific, Waltham, MA, USA) to analyze the elemental composition of PS_46k_-N=CH-PMMA_23k_ and PS_40k_-N=CH-PMMA_20k_ polymer thin films before and after etching; samples were prepared by thermal annealing at 230 °C for 30 min on silicon substrates modified with a random copolymer Mat67 (F_St_ = 67%). Film thicknesses were measured using a Filmetrics F20-UV spectroscopic reflectometer (KLA Corporation, San Diego, CA, USA), which operates over a wavelength range of 190–1100 nm and offers a thickness measurement range of 5 nm to 40 μm (assuming n = 1.46), with a resolution of 0.02 nm. Small-angle X-ray scattering (SAXS) was used to investigate the microphase-separated structures of PS-N=CH-PMMA and PS-*b*-PMMA in the bulk state; SAXS profiles were plotted as the scattering intensity (I) versus the scattering vector (q*), and the domain spacing (*L*_0_) of the cylindrical structures was calculated using the Bragg equation (*L*_0_ = 2π/q*), where q* corresponds to the position of the primary scattering peak. The SAXS samples were prepared as transparent discs with an 8 mm diameter using a hydraulic tablet press (HY-12) and annealed at 230 °C for 30 min; experiments were carried out at room temperature on the BL16B1 beamline of the Shanghai Synchrotron Radiation Facility. Morphological analyses were performed using a Zeiss Gemini 300 scanning electron microscope (Carl Zeiss AG, Oberkochen, Germany) without gold sputtering. The Inductively Coupled Plasma (ICP) system used in this study was Plasma 100, manufactured by Oxford Instruments (Abingdon, Oxfordshire, UK). Focused ion beam transmission electron microscopy (FIB-TEM) analyses were carried out by Nanjing Pan Quan Electronic Technology Co., Ltd. (Nanjing, China); a protective layer of hafnium oxide was deposited on the sample surfaces before FIB preparation. The samples were fabricated using a Thermo Fisher Scientific Helios Dual-beam system (Thermo Fisher Scientific, Waltham, MA, USA), and TEM imaging was conducted on a Talos microscope (Thermo Fisher Scientific, Waltham, MA, USA) operating at 200 kV in bright-field mode.

## 3. Results and Discussion

### 3.1. Synthesis and Characterization of BCPs

**Figure 1:**
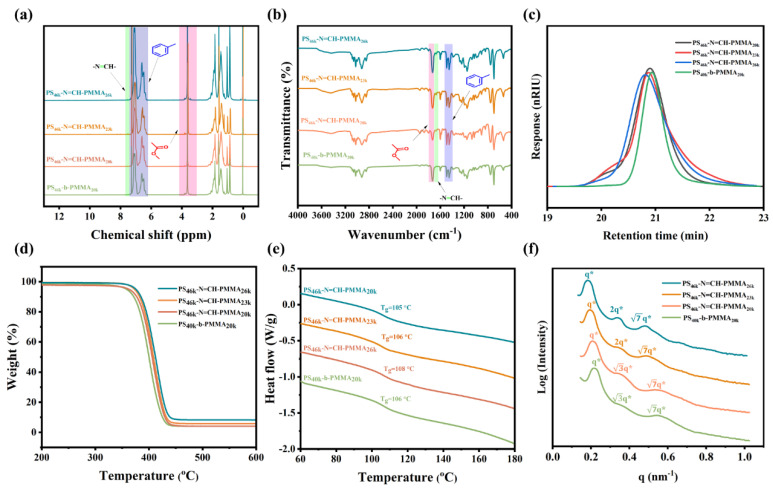
Characterization of PS-N=CH-PMMA BCPs and conventional PS-*b*-PMMA. (**a**) ^1^H NMR spectra, (**b**) FTIR spectra, (**c**) GPC traces, (**d**) TGA curves, (**e**) DSC curves, and (**f**) SAXS profiles.

Acid-responsive PS-N=CH-PMMA BCPs were synthesized Via a Schiff base reaction between amine-terminated polystyrene (PS-NH_2_) and aldehyde-terminated PMMA (PMMA-CHO). In contrast, the conventional PS_40k_-*b*-PMMA_20k_ was prepared by atom transfer radical polymerization (ATRP), as illustrated in Scheme 1a. The synthetic routes for PMMA-OH, PMMA-CHO, and PMMA-Br are illustrated in Figure S1. PMMA-OH homopolymers were synthesized Via ATRP using 2-hydroxyethyl 2-bromo-2-methylpropanoate as the initiator to introduce well-defined hydroxyl end groups, which were subsequently converted to aldehyde functionalities via Steglich esterification [54]. Additionally, PMMA-Br was synthesized Via ATRP using ethyl α-bromophenylacetate as the initiator. The successful synthesis of the PMMA-based macromolecules (PMMA-OH, PMMA-CHO, and PMMA-Br) was confirmed by ^1^H NMR spectroscopy and GPC, as shown in Figures S2 and S3. Scheme 1b illustrates the process of thin-film self-assembly followed by wet etching for PS-N=CH-PMMA BCPs. The process includes three key steps: spin-coating of the BCP, thermal annealing to induce BCP microphase separation, and selective removal of the PMMA domain by immersion in glacial acetic acid. The successful preparation of PS-N=CH-PMMA BCPs and PS_40k_-*b*-PMMA_20k_ was verified by ^1^H NMR spectroscopy (Figure 1a). The aromatic protons of PS appeared at 6.3–7.3 ppm, while the methoxy (–OCH_3_) and methyl (–CH_3_) protons of PMMA showed peaks around 3.6 ppm and 0.8–1.2 ppm, respectively. In particular, the PS-N=CH-PMMA spectrum showed a unique resonance at 7.53 ppm, attributed to the imine (–N=CH–) linkage introduced by the Schiff base. This spectral feature confirms the successful incorporation of the Schiff base and distinguishes PS-N=CH-PMMA from the conventional PS_40k_-*b*-PMMA_20k_. FTIR spectra of PS-N=CH-PMMA (Figure 1b) also confirmed the presence of the Schiff base linkage. PS-N=CH-PMMA exhibits a distinct absorption band at ~1620 cm^−1^ (the imine C=N stretching vibration, highlighted in green in Figure 1b), absent in the PS-b-PMMA spectrum. This contrast highlights the successful incorporation of the Schiff base linkage into the copolymer backbone. Furthermore, all samples exhibited characteristic bands at ~1728 cm^−1^ (carbonyl C=O stretch of PMMA, pink) and ~1490 cm^−1^ (aromatic C–H bend of PS, purple). The molecular weights and polydispersity indices (PDI) were determined by GPC (Figure 1c). All samples exhibited monomodal elution profiles, confirming the successful formation of well-defined BCPs. The GPC trace of the reference PS_40k_-*b*-PMMA_20k_ displayed a single, symmetric peak, indicating a narrow molecular weight distribution. In contrast, the PS-N=CH-PMMA series exhibited a minor shoulder peak on the high molecular weight side. This shoulder is attributed to the commercial PS-NH_2_ precursor used in the stepwise coupling reaction, which itself shows a small shoulder in its original GPC trace, likely due to minor chain-chain coupling or compositional heterogeneity during its anionic polymerization. Consequently, the PS-N=CH-PMMA samples displayed slightly higher molecular weights (M_n_ ≈ 70.4 kg/mol) than the reference PS_40k_-*b*-PMMA_20k_ (M_n_ ≈ 65.5 kg/mol), along with a modestly increased PDI (1.12 Vs. 1.09), as summarized in Table 1. This broadening is attributed to variations in the coupling efficiency inherent to the stepwise synthesis process. Moreover, a subtle shoulder on the high molecular weight side in the GPC trace of the PS-N=CH-PMMA series is attributed to the commercial PS-NH_2_ precursor used in the imine coupling reaction, which itself displays a similar feature (Figure S4), likely resulting from minor chain–chain coupling or molecular weight heterogeneity introduced during anionic polymerization [55]. Because the self-assembly of conventional PS-*b*-PMMA typically requires thermal annealing, we evaluated the thermal stability of PS-N=CH-PMMA BCPs. DSC and TGA were conducted to characterize the thermal behavior of the BCPs. The TGA curve (Figure 1d) shows that both copolymers begin to degrade at around 250 °C thermally. These results indicate that incorporating the Schiff base linkage does not significantly alter the polymer’s thermal stability. As shown in Figure 1e, PS-N=CH-PMMA has a glass transition temperature (T_g_) of approximately 105 °C, which is in close agreement with that of PS-*b*-PMMA. The similar T_g_ and decomposition temperature suggest that PS-N=CH-PMMA BCPs have sufficient thermal stability to withstand standard high-temperature annealing protocols used in DSA. The domain spacing (*L*_0_) is an essential parameter for self-assembly in confined templates and can be determined from small-angle X-ray scattering (SAXS) data. SAXS profiles (Figure 1f) for both PS-N=CH-PMMA BCPs and PS_40k_-*b*-PMMA_20k_ show a series of diffraction peaks at q*, $\sqrt{3}$q*, 2q*, and $\sqrt{7}$q*, corresponding to the first and higher order reflections of a hexagonally packed cylindrical morphology. The observed scattering pattern confirms that both BCPs adopt a well-ordered cylindrical morphology, in which PMMA forms vertically oriented cylinders hexagonally packed within a continuous PS matrix. The domain spacings of the cylindrical morphology were calculated using Bragg’s law (*L*_0_ = 2π/q*), with values in the range of 31.1–34.3 nm for PS-N=CH-PMMA BCPs and approximately 27.9 nm for PS_40k_-*b*-PMMA_20k_. The larger *L_0_* of PS-N=CH-PMMA BCPs is attributed to their higher molecular weight. This observation is consistent with previously reported trends correlating higher molecular weight with larger domain spacing [56]. Furthermore, Table 1 shows that the *L*_0_ of PS-N=CH-PMMA BCPs can be tuned by adjusting the PMMA block molecular weights, providing flexibility for targeted self-assembly and DSA processes. To investigate the effect of the Schiff base linkage on interfacial properties, PS_46k_-N=CH-PMMA_23k_ and PS_40k_-*b*-PMMA_20k_ were selected for comparison, given their nearly identical ƒ_St_. Contact angle measurements using water and diiodomethane (CH_2_I_2_) were performed to assess surface energy, as presented in Figure S8. The surface energies of the films were subsequently calculated using the Fowkes method, which evaluates material interactions with polar and nonpolar liquids [57]. PS_46k_-N=CH-PMMA_23k_ exhibited contact angles of 81.2° with water and 33.8° with CH_2_I_2_, while PS_40k_-*b*-PMMA_20k_ showed comparable values of 81.8° and 32.7°, respectively (Table S1). The corresponding surface energies were calculated to be 43.8 and 44.1 mN m^−1^, indicating negligible differences between the two BCP films. These results suggest that the incorporation of the Schiff base bond into the polymer backbone does not significantly alter the material’s surface energy.

### 3.2. Morphological Analysis of BCP Films

**Figure 2:**
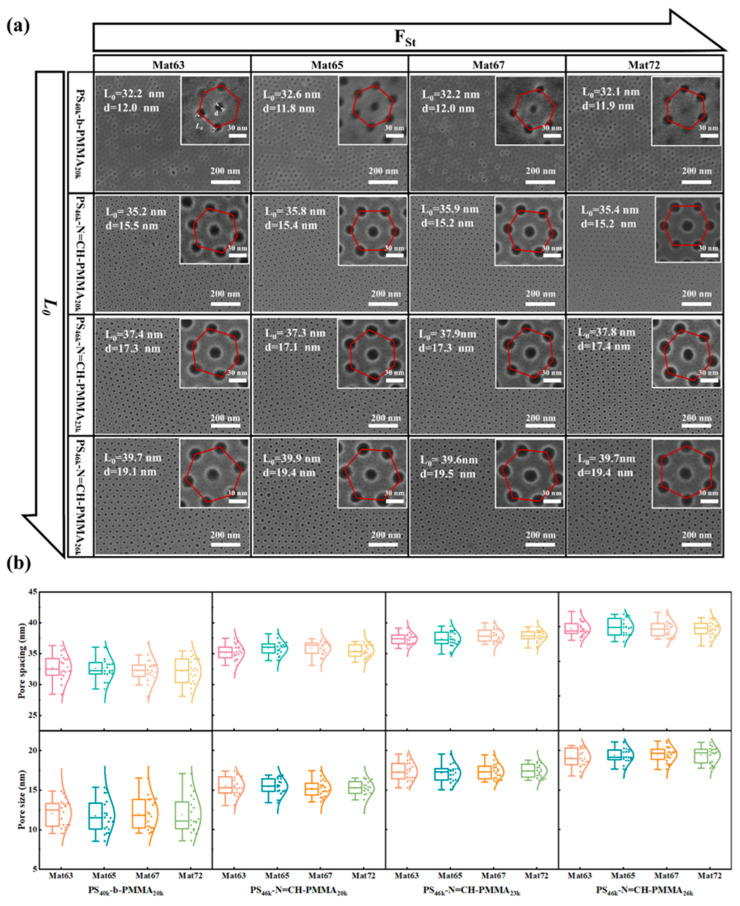
Morphological characterization of PS-N=CH-PMMA and conventional PS-*b*-PMMA BCP thin films. (**a**) Top view SEM images of nanopatterns formed by PS_46k_-N=CH-PMMA_20k_, PS_46k_-N=CH-PMMA_23k_, PS_46k_-N=CH-PMMA_26k_ (three bottom rows), and PS_40k_-*b*-PMMA_20k_ (top row) on Mat-modified substrates with various F_St_ after selective removal of PMMA by acetic acid wetting etching. (**b**) The statistical analysis of size (d) and spacing (*L*_0_) of BCP-derived pores on different Mats, n = 20.

Surface modification of substrate using random copolymer mats or polymer brushes plays a critical role in directing the orientation of microdomains and balancing interfacial energy in PS-*b*-PMMA thin films [58]. Prior studies have shown that random copolymer mats (poly (styrene-co-methyl methacrylate-co-glycidyl methacrylate)) can induce perpendicular orientation of cylindrical microdomains in PS-*b*-PMMA thin films upon thermal treatment [59]. Expanding on that, we systematically investigated the self-assembly behavior of the synthesized acid-responsive PS-N=CH-PMMA BCPs, along with the conventional PS-*b*-PMMA, on silicon substrates modified with a synthesized mat of measured polystyrene molar fractions (F_St_ = 63%, 65%, 67%, and 72%). The mats were synthesized Via free radical solution polymerization (Figure S5), in which the feed monomer ratios were systematically varied to precisely tune the surface energy of the substrate. These random copolymers, typically composed of PS and PMMA segments with a small fraction of anchoring units, serve as Mat layers to establish near-neutral interfacial interactions with both blocks, thereby facilitating the formation of perpendicularly oriented BCP nanodomains under thermal annealing conditions [60,61]. The GPC and ^1^H NMR analyses (Figures S6 and S7), along with the corresponding molecular characteristics and measured film thicknesses, are summarized in Table 2. Figure 2a presents top-view SEM images of the resulting self-assembled morphologies upon thermal treatment for 30 min at 230 °C. Tested BCPs are conventional PS_40k_-*b*-PMMA_20k_ and three Schiff base-linked PS_46k_-N=CH-PMMA_n_ BCPs with different PMMA block lengths (n = 20k, 23k, 26k). All BCPs form perpendicularly oriented cylindrical morphologies on the full range of neutral mats tested (Mat63–Mat72), indicating good interfacial adaptability of the system and effective reduction or neutralization of interfacial interactions between the BCPs and the substrate. Despite their similar self-assembled morphologies, noticeable differences in pattern fidelity emerged after wet etching. Although the structural integrity of the PS_40k_-*b*-PMMA_20k_ films was largely retained following acetic acid treatment, numerous pores appeared closed or blind, indicating incomplete removal of the PMMA domains and insufficient etching depth through the entire film. This restricted etching selectivity is due to the inherent acid stability of the covalent bond between blocks and the fact that there are no cleavable units in the conventional BCP design. In contrast, all three PS-N=CH-PMMA materials produce distinct nanopores following immersion in glacial acetic acid for 5 min. When the molecular weight of the block of PMMA rose from 20k to 26k, the pore spacing (*L*_0_) values increased from 35.2 nm to 39.7 nm, and the corresponding pore diameter (d) increased from 15.5 nm to 19.5 nm. This selectivity is enabled by acid-catalyzed hydrolysis of the imine (-N=CH-) bonds between PS and PMMA blocks, allowing complete PMMA dissolution while preserving the structural integrity of the PS mask.

The statistical analysis of the SEM images enables accurate determination of the d and *L*_0_, as illustrated in Figure 2b. The box plots show a clear pore uniformity difference between the two BCP variants. Whereas conventional PS_40k_-*b*-PMMA_20k_ exhibits a relatively broad distribution in pore diameter, the acid-responsive PS-N=CH-PMMA variants present significantly narrower distributions, indicating improved uniformity of the etched nanopatterns. In particular, *L*_0_ negatively correlates with the F_St_ of the BCPs. At the same time, d shows a positive correlation, demonstrating that both *L*_0_ and d can be precisely tuned by adjusting the length of the PMMA-CHO block. This synthetic flexibility offers a new strategy for decoupling polymer, allowing systematic tuning of structural dimensions.

### 3.3. Wet Etching Mechanism of the PS-N=CH-PMMA Film

**Figure 3:**
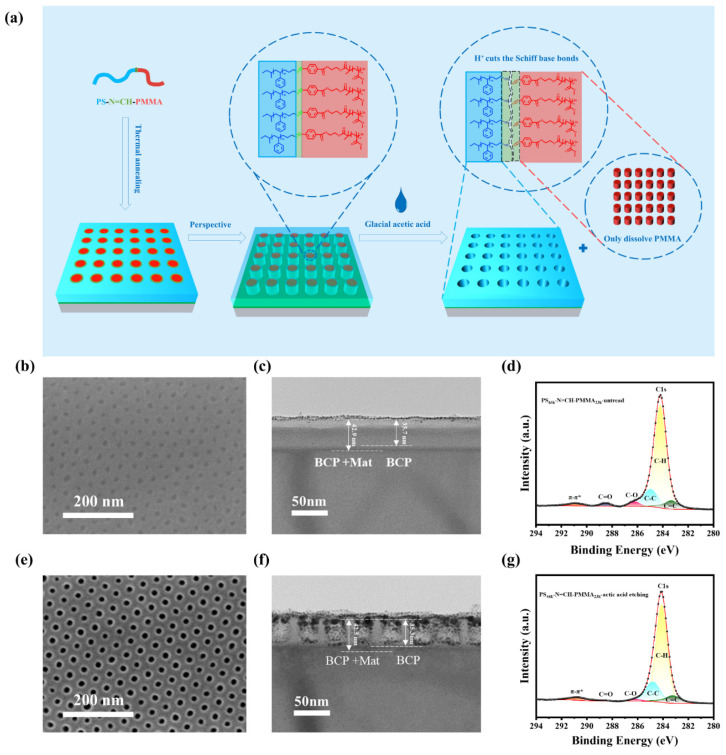
Mechanistic analysis of wet etching in PS-N=CH-PMMA thin films. (**a**) Schematic illustration of the wet etching mechanism for nanoporous films formed by the thin film self-assembly of PS-N=CH-PMMA BCPs. (**b**) SEM images of the morphology, (**c**) FIB-TEM image of the corresponding cross-sectional morphology, and (**d**) XPS C1s spectra of PS-N=CH-PMMA nanoporous film before wet etching. (**e**) SEM images of the morphology, (**f**) the FIB-TEM image of the corresponding cross-sectional morphology, and (**g**) the XPS C1s spectra of the PS-N=CH-PMMA assembly film after wet etching.

To investigate the wet etching mechanism of Schiff base bond-functionalized BCPs, we conducted a study with PS_46k_-N=CH-PMMA_23k_ as a representative material. As illustrated in Figure 3a, the dynamic Schiff base linkages (-N=CH-) located at the PS/PMMA interfaces undergo selective acid-catalyzed cleavage in the presence of protons (H^+^) from glacial acetic acid, a mechanism well-documented for imine-based systems [62,63]. Concurrently, glacial acetic acid acts as a polar solvent that preferentially dissolves the polar PMMA domains while leaving the hydrophobic PS matrix intact, owing to their inherent solubility contrast [64]. The simultaneous bond cleavage and selective solvation ensure the formation of well-defined nanoporous structures with minimal lateral deformation, achieving vertically aligned nanopores with high fidelity. Top-view SEM images of self-assembly morphology before and after wet etching are shown in Figure 3b,e, and a significant change in contrast was observed in the SEM images. The efficient removal of PMMA could be evidenced by forming a highly ordered nanoporous structure and well-preserved pattern integrity. Moreover, the cross-section of the self-assembly morphology of the BCP thin film was characterized. The PS and PMMA phases could not be distinguished before wet etching due to the lower contrast between PS and PMMA, as shown in Figure 3c. Intriguingly, the cross-sectional TEM image in Figure 3f reveals that the holes are fully opened after the selective removal of the PMMA phase with acid, coupled with gold coating treatment. Meanwhile, the film thickness is 42.5 nm, which remains consistent with the film before etching (42.9 nm). These results confirm that wet etching successfully cleaves the Schiff base bonds at the PS/PMMA interface, enabling the selective removal of the PMMA while preserving the PS matrix with no significant influence on the overall film thickness. In particular, the remaining layer with a thickness of 7.2 nm corresponds to the Mat67 layer, which acts as a neutral layer on the substrate. X-ray photoelectron spectroscopy (XPS) was further employed to investigate the chemical changes in PS_46k_-N=CH-PMMA_23k_ before and after wet etching, as previously reported in similar studies [33]. The C1s spectra (Figure 3d,g) reveal significant changes in the chemical composition. The spectrum shows a prominent peak at 287.6 eV, attributed to C=O bonds, confirming the presence of PMMA before wet etching. Additional peaks at 284.8 and 285.6 eV correspond to C-C and C-H bonds originating from PS and PMMA. The C=O peak is almost completely diminished after wet etching, as demonstrated in Figure 3g, indicating the successful removal of PMMA from the BCP. Peaks at 284.8 and 285.6 eV remain dominant and unchanged, confirming the preservation of the PS matrix during the etching process. These results further support the proposed mechanism that acetic acid cleaves the Schiff base bonds at the PS/PMMA interface and selectively dissolves the PMMA phase during the etching process while the PS phase remains intact.

### 3.4. Graphoepitaxial DSA of PS-N=CH-PMMA BCPs

**Figure 4:**
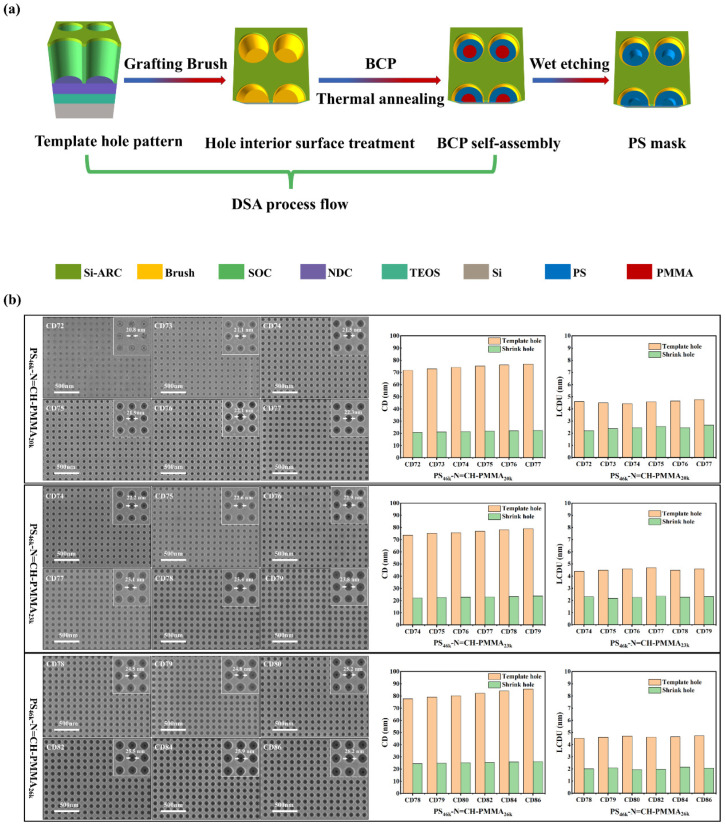
DSA process flow and shrink-hole patterning morphological analysis of PS-N=CH-PMMA BCPs with varying PMMA block lengths. (**a**) Schematic process flow of shrink-hole patterning Via DSA and wet etching using PS-N=CH-PMMA BCPs. (**b**) SEM images of shrink-hole patterns in different template holes. The bar charts on the right present the statistical analysis of critical dimension (CD) and local critical dimension uniformity (LCDU) for both the origin template holes and the DSA shrinkage holes, n = 20.

DSA with PS-*b*-PMMA has attracted considerable interest in forming nanostructures, especially in pattern-shrinking applications [65]. Conventional BCP systems often leave residual material on the template surface due to overfilling the template holes, which cannot be entirely removed by the wet etching [66]. To effectively tackle this problem, we adopted an underfilling approach in this study, depositing an amount of BCP less than the hole’s volume. This strategy facilitates a more accurate evaluation of the wet etching behavior in DSA processes. To evaluate the influence of PMMA block length on shrink-hole patterning performance, three PS-N=CH-PMMA BCPs with identical PS block lengths (M_n_ = 46k) and varying PMMA block lengths (M_n_ = 20k, 23k, 26k) were investigated. Figure 4a schematically illustrates the overall shrink-hole fabrication workflow, which includes two main steps: (i) directed self-assembly of PS-N=CH-PMMA BCPs inside cylindrical templates Via grafting of hydroxyl-terminated poly (styrene-co-methyl methacrylate) (P20255a) random copolymers and thermal annealing, and (ii) subsequent wet etching by selectively removing the PMMA domains to generate PS masks. Top-view SEM images in Figure 4b reveal the resulting shrink-hole morphologies across a series of template holes with varying critical dimensions (CDs, defined here as the pore diameter). All three BCP systems demonstrated successful confinement-induced assembly within cylindrical hole templates, yielding well-ordered shrink holes across various template CDs. PS_46k_-N=CH-PMMA_23k_ and PS_46k_-N=CH-PMMA_26k_ enabled effective contact hole shrinkage, achieving a hole opening yield approaching 100% within the CD range of 74–86 nm. Defect-free and uniform shrink-hole patterns were obtained, indicating a favorable alignment between the template CD and the BCPs’ domain spacing (*L*_0_). As *L*_0_ increased from 35.9 to 39.6 nm, the optimal template CD range shifted to larger values, broadening the processing window toward higher critical dimensions (Figure S9). Statistical analysis of shrink-hole CD and local critical dimension uniformity (LCDU), as shown in the bar charts on the right side of Figure 4b, further confirms the patterning quality. In all cases, the CD and LCDU of the shrink holes were significantly reduced compared to those of the original template, proving the effectiveness of the contact hole shrinkage process. As the PMMA block length increased to 26k, the shrink-hole CDs became slightly larger, consistent with an increased overall domain spacing, which favors assembly in templates with larger CDs. These results demonstrate that fine-tuning the PMMA block length provides a practical route to optimize shrink-hole fidelity and uniformity in DSA patterning. PS-N=CH-PMMA BCPs combine high etch selectivity, low LCDU, and robust process compatibility, making them well-suited for advanced sub-30 nm patterning technologies.

### 3.5. Pattern Transfer of DSA Shrink-Hole Structures into TEOS Layer

**Figure 5:**
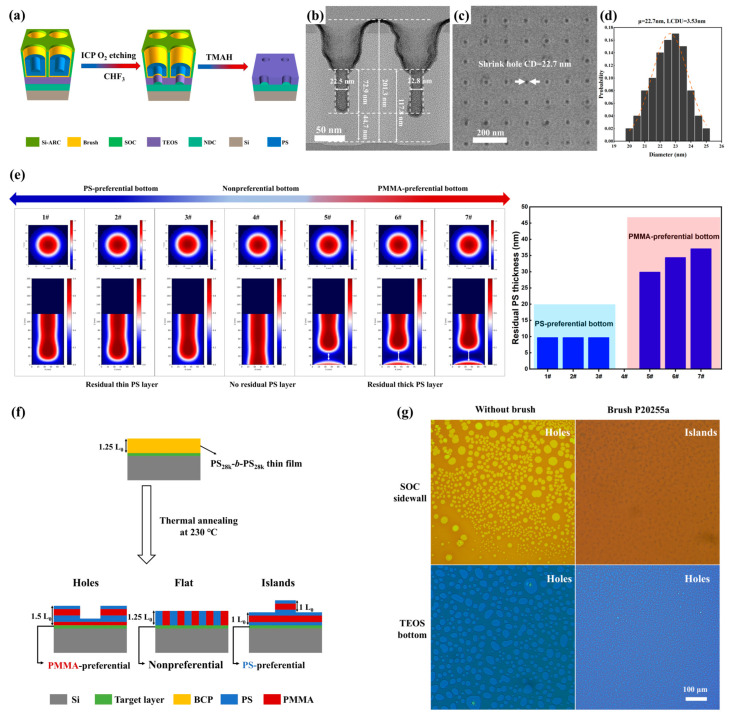
Pattern Transfer of DSA Shrink-Hole using acid-responsive PS-N=CH-PMMA. (**a**) Process flow for transferring PS shrink-hole patterns into the TEOS layer using DSA and wet etching of PS-N=CH-PMMA. (**b**) Cross-sectional FIB-TEM image of shrink-hole patterns formed by PS_46k_-N=CH-PMMA_23k_ after wet etching. (**c**) Top-view SEM image of the corresponding hole array transferred into the TEOS layer using PS_46k_-N=CH-PMMA_23k_. (**d**) Histograms of the statistical distribution of the cylinder diameter of PS_46k_-N=CH-PMMA_23k_ after pattern transfer. (**e**) Ohta-Kawasaki model simulations of vertical phase separation morphologies of PS-N=CH-PMMA BCP inside hole templates, with fixed PS-preferential sidewalls and varying bottom surface affinities. (**f**) Schematic illustration of PS_28k_-*b*-PMMA_28k_ films forming lamella with a thickness of 1.25 *L*_0_ that were thermally annealed at 230 °C on substrates exhibiting different affinities for PS and PMMA. (**g**) Optical micrographs of thin films of 37.5 nm-thick lamella PS_28k_-*b*-PMMA_28k_ (*L*_0_ = 30 nm) deposited on a mimicked SOC sidewall and TEOS bottom surfaces before and after the application of a brush layer (P20255a).

To further assess the applicability of PS-N=CH-PMMA BCPs in high-resolution pattern transfer for semiconductor manufacturing, PS_46k_-N=CH-PMMA_23k_ was selected as a representative sample. The DSA shrink-hole pattern transfer process is schematically illustrated in Figure 5a and comprises three main steps: (i) plasma etching with ICP O_2_ to remove the residual PS and underlying brush layers; (ii) CHF_3_ etching to transfer the pattern into the TEOS layer; and (iii) TMAH treatment to eliminate any remaining overlying layers and PS mask. The corresponding guide template structure used for PS_46k_-N=CH-PMMA_23k_ features a critical dimension (CD) of 75 nm. The cross-sectional TEM image (Figure 5b) reveals a narrow, vertically elongated neck profile with a consistent diameter of 22.5 nm and steep sidewalls attributed to the selective removal of the PMMA domain Via acetic acid rinsing. The neck extends approximately 72.9 nm in height, indicating effective vertical dimensional control. The remaining polymer thickness, including the residual PS and underlying brush layers, is approximately 44.7 nm. Given the high etching selectivity of PS-N=CH-PMMA and the preserved PS mask, we evaluated the material’s pattern transfer capability to the TEOS layer Via combined wet and dry etching. As shown in Figure 5c, the shrink-hole patterns were successfully transferred onto the TEOS layer with minimal distortion. Quantitative analysis (Figure 5d) remained nearly unchanged in hole diameter from 22.6 to 22.7 nm after pattern transfer, with LCDU of 3.53 nm. The high-quality vertical selectivity achieved through wet etching significantly improved the efficiency of subsequent dry etching, facilitating the complete removal of the residual PMMA.

To elucidate the underlying mechanism responsible for PS residue formation at the bottom of the shrink-hole templates, we performed Ohta-Kawasaki theoretical simulations to examine the vertical phase separation behavior of PS-N=CH-PMMA BCPs within cylindrical confinement (Figure 5e). In all simulations, the sidewalls were set to be PS-preferential, while the bottom surface affinity was systematically varied from PS-preferential (samples 1#–3#) to neutral (4#) and further to PMMA-preferential (5#–7#). As shown in Figure 5e, the PS domain consistently wetted the sidewalls across all conditions due to the imposed boundary preference. However, the vertical distribution of PS and PMMA phases near the bottom interface strongly depended on the bottom surface affinity. The polymer chains reorganized for PS-preferential or neutral bottoms to minimize unfavorable interactions, leading to either a thin PS residue layer or complete phase separation without residue. In contrast, when the bottom surface was PMMA-preferential, the PMMA block was preferentially drawn toward the bottom, displacing the PS chains upward and resulting in a thick PS accumulation at the interface. The bar chart on the right summarizes the residual PS layer thickness extracted from the simulation outputs. A clear trend was observed: as the bottom surface affinity shifted from PS- to PMMA-preferential, the residual PS layer thickness increased significantly. These results confirm that interfacial wetting asymmetry, specifically the coexistence of PS-preferring sidewalls and a PMMA-preferring bottom surface, is a critical driver of vertical PS enrichment and the formation of undesired residual layers.

A previous study employed a lamella-forming BCP to investigate the preferential wetting behavior of polymer brush-modified template hole sidewalls and bottoms by analyzing the surface morphology of the resulting self-assembled thin films [67]. Building on this method, we evaluated the interfacial wetting characteristics of the polymer brush P20255a on SOC and TEOS substrates, which were used to simulate the sidewalls and bottom surfaces of template holes, respectively. As shown in Figure 5f, PS_28k_-*b*-PMMA_28k_ lamella-forming films were deposited on the substrates modified with P20255a brush. The corresponding surface morphologies before and after brush treatment are presented in Figure 5g. The results (Table S2) confirm that the P20255a brush induces preferential PS wetting on SOC sidewalls and preferential PMMA wetting on the TEOS bottom surface, explaining the residual PS layer observed at the template bottom (Figure 5b). The observed difference in interfacial wetting behavior is likely attributed to variations in brush grafting density, as the higher surface energy and increased roughness of SOC sidewalls facilitate stronger physical adsorption and enhanced chain mobility, thereby promoting denser grafting.

## 4. Conclusions

In summary, we have successfully synthesized an acid-responsive Schiff base-linked cylindrical BCP, PS-N=CH-PMMA. This material self-assembles into well-ordered cylindrical nanodomains and exhibits excellent etching selectivity for the PMMA block, enabling the formation of high-fidelity PS mask patterns during wet etching. The wet etching mechanism—cleaving the inter-block imine linkages and dissolving PMMA—allows pattern formation without a separate PMMA degradation step (e.g., no UV exposure required) and produces a vertically oriented PS mask of full film thickness. The self-assembled domain spacing can be tuned to match the dimensions of target-specific template holes by choosing appropriate PMMA-CHO block molecular weights. Graphoepitaxial DSA for template hole shrinkage yields nearly 100% shrinkage of the hole opening over an expanded process window. This high etch contrast between blocks simplifies subsequent pattern transfer steps: using an underfilled approach and O_2_ ICP dry etching, the PS mask can be transferred into the underlying TEOS layer with minimal CD loss and excellent CD uniformity. These results highlight PS-N=CH-PMMA as a promising material for next-generation DSA lithography, delivering enhanced etch selectivity and process flexibility over conventional PS-*b*-PMMA.

## 5. Patents

Patents related to this work are currently pending under application number 202311567642.5.

## Figures and Tables

**Table 1:** Molecular weight (M_n_ BCP) of the BCP, molecular weight (M_n_ PMMA) of the PMMA, Polydispersity Index (PDI), PS volume fraction (ƒ_St_), and domain spacing (*L*_0_) of all BCPs.

Sample ^a^	M_n BCP_ ^b^ (kg/mol)	M_n PMMA_ ^b^ (kg/mol)	PDI ^b^	ƒ_St_ ^c^	*L*_0 SEM_ ^d^ (nm)	*L*_0 SAXS_ ^e^ (nm)	Film Thickness ^f^ (nm)
PS_46k_-N=CH-PMMA_20k_	65.5k	20.1k	1.12	0.725	35.9	31.1	32.8
PS_46k_-N=CH-PMMA_23k_	67.7k	23.3k	1.12	0.703	37.9	32.8	35.3
PS_46k_-N=CH-PMMA_26k_	70.4k	26.1k	1.12	0.674	39.6	34.3	37.5
PS_40k_-*b*-PMMA_20k_	60.0k	20.5k	1.09	0.702	32.2	27.9	30.3

^a^ PS_x_-N = CH-PMMA_y_ refers to an acid-responsive BCP with M_n_ (PS) = x kg mol^−1^ and M_n_ (PMMA) = y kg mol^−1^. ^b^ Determined by GPC in THF against PS standards. ^c^ Determined by ^1^H NMR spectroscopy in CDCl_3_. ^d^ Determined by SEM. ^e^ The position of the first-order peak in the SAXS profile. ^f^ The film thicknesses of various BCP samples were measured using a Filmetrics F20-UV spectroscopic reflectometer.

**Table 2:** Molecular weight, polydispersity index (PDI), and PS molar fraction of the synthesized Mats.

Sample ^a^	M_n_ ^b^ (kg/mol)	PDI ^b^	F_St_ ^c^ (%)	Film Thickness ^d^ (nm)
Mat63	24.4	1.55	63.2	6.4
Mat65	25.2	1.57	65.4	6.5
Mat67	28.5	1.55	67.7	7.3
Mat72	28.1	1.56	72.3	6.9

^a^ Mat-x refers to a random copolymer with x mol% PS units. ^b^ Determined by GPC in THF against PS standards. ^c^ Determined by ^1^H NMR spectroscopy in CDCl_3_. ^d^ Determined by Filmetrics F20-UV spectroscopic reflectometer.

## Data Availability

The data that support the findings of this study are available on request from the corresponding author.

## Supplementary Materials

The following supporting information can be downloaded at https://www.mdpi.com/article/10.3390/nano15201571/s1: Figure S1: Synthesis scheme of homopolymers: PMMA-OH, PMMA-CHO, and PMMA-Br; Figure S2: ^1^H NMR spectra of the polymers: (a) PMMA_20k_-OH, PMMA_23k_-OH, and PMMA_26k_-OH, (b) PMMA_20k_-CHO, PMMA_23k_-CHO, and PMMA_26k_-CHO, and (c) PMMA_20k_-Br and the BCP PS_40k_-*b*-PMMA_20k_; Figure S3: GPC spectra of polymers: (a) PMMA-OH homopolymers, (b) PMMA-CHO homopolymers, and (c) homopolymer PMMA_20k_-Br and BCP PS_40k_-*b*-PMMA_20k_; Figure S4: GPC spectrum of commercial amino-terminated polystyrene (PS_46k_-NH_2_); Figure S5: Synthesis scheme of random copolymer mats; Figure S6: GPC spectra of the random copolymer mats; Figure S7: ^1^H NMR spectra of the random copolymer mats; Figure S8: Contact angle measurements for PS_46k_-N=CH-PMMA_23k_ and PS_40k_-*b*-PMMA_20k_ were performed using water and diiodomethane; Figure S9: Process window analysis of different PS-N=CH-PMMA samples across the template critical dimension (CD) range; Table S1: Contact angle measurement and surface energy calculation of PS_46k_-N=CH-PMMA_23k_ and PS_40k_-*b*-PMMA_20k_; Table S2: Summary of wetting behaviors of bottom TEOS and sidewall SOC surfaces.
