# Supplementary material for: Directed Self-Assembly of an Acid-Responsive Block Copolymer for Hole-Shrink Process and Pattern Transfer
Source: Nanomaterials (Basel). 2025 Oct 16;15(20):1571. doi: 10.3390/nano15201571 (PMC12566743; doi:10.3390/nano15201571)
Supplement: Supplementary file 1 [file nanomaterials-15-01571-s001.zip › nanomaterials-3911277-supplementary.pdf]

Directed Self-Assembly of an Acid-Responsive Block Copolymer for Hole-Shrink Process and Pattern Transfer

Jianghao Zhan, Jiacheng Luo, Zixin Zhuo, Caiwei Shang, Zili Li, Shisheng Xiong**

Center of Micro-Nano System, School of Information Science and Technology, Fudan University, Shanghai, 200438, China

Corresponding Author

* E-mail address: lizili@fudan.edu.cn; sxiong@fudan.edu.cn.

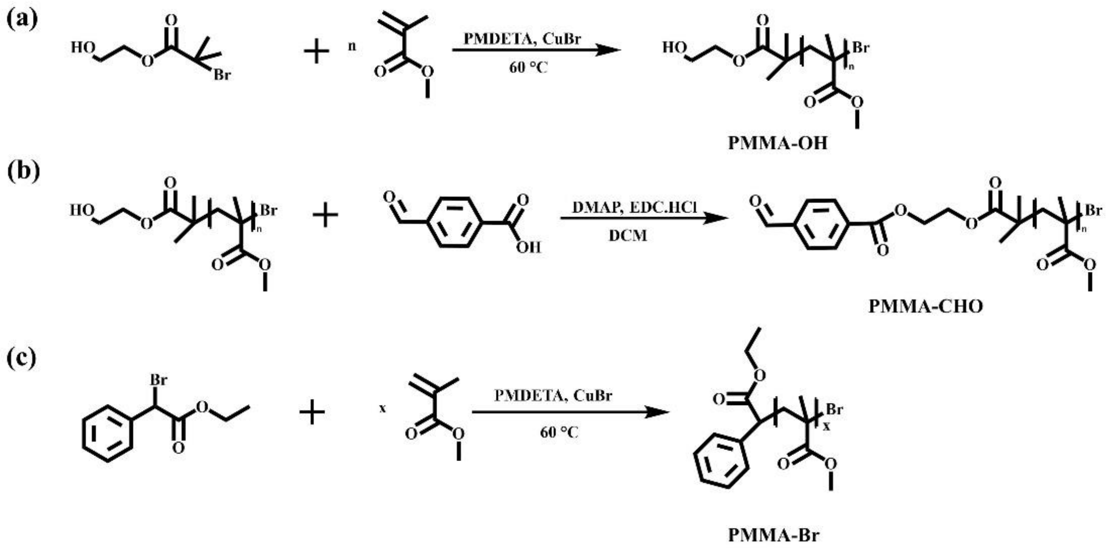

Figure S1. Synthesis scheme of homopolymers: PMMA-OH, PMMA-CHO, and PMMA-Br.

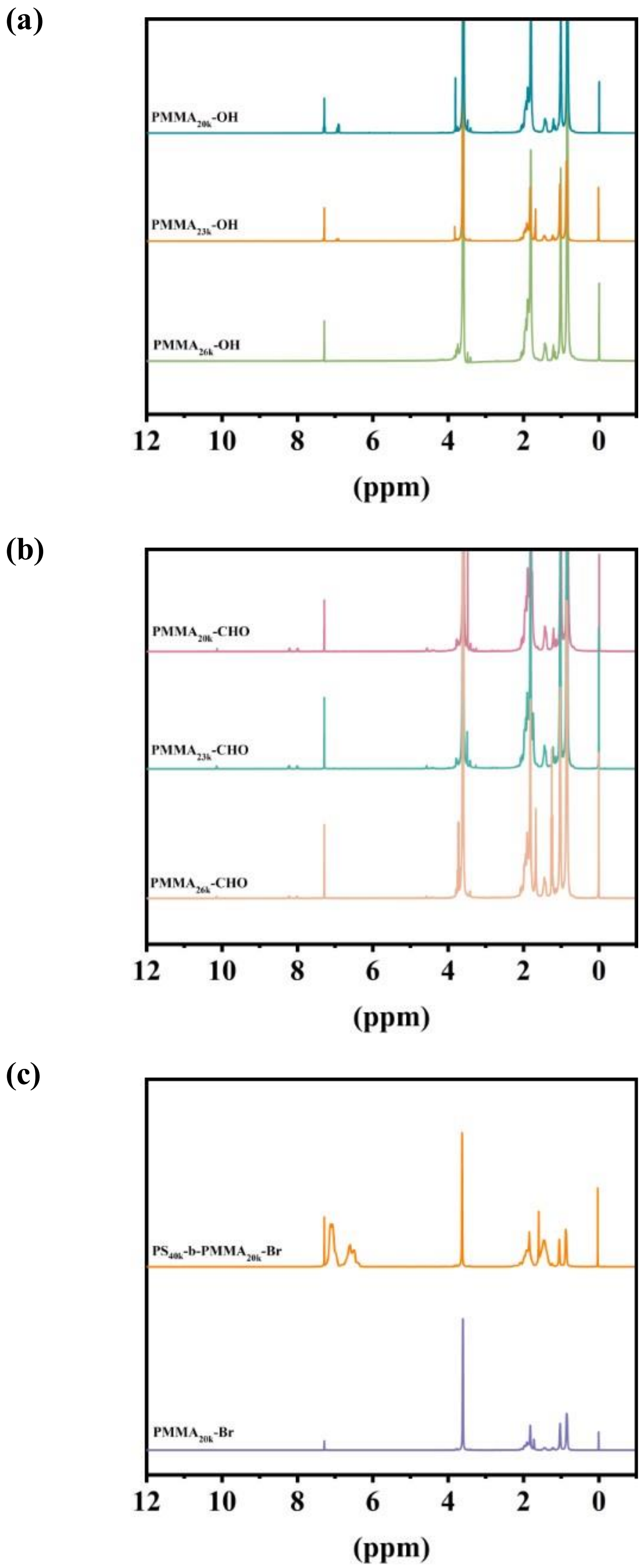

Figure S2. ^{1}H NMR spectra of the polymers: (a) $PMMA_{20k}$-OH, $PMMA_{23k}$-OH, and $PMMA_{26k}$-OH, (b) $PMMA_{20k}$-CHO, $PMMA_{23k}$-CHO, and $PMMA_{26k}$-CHO, and (c) $PMMA_{20k}$-Br and the block copolymer PS_{40k}-*b*-$PMMA_{20k}$.

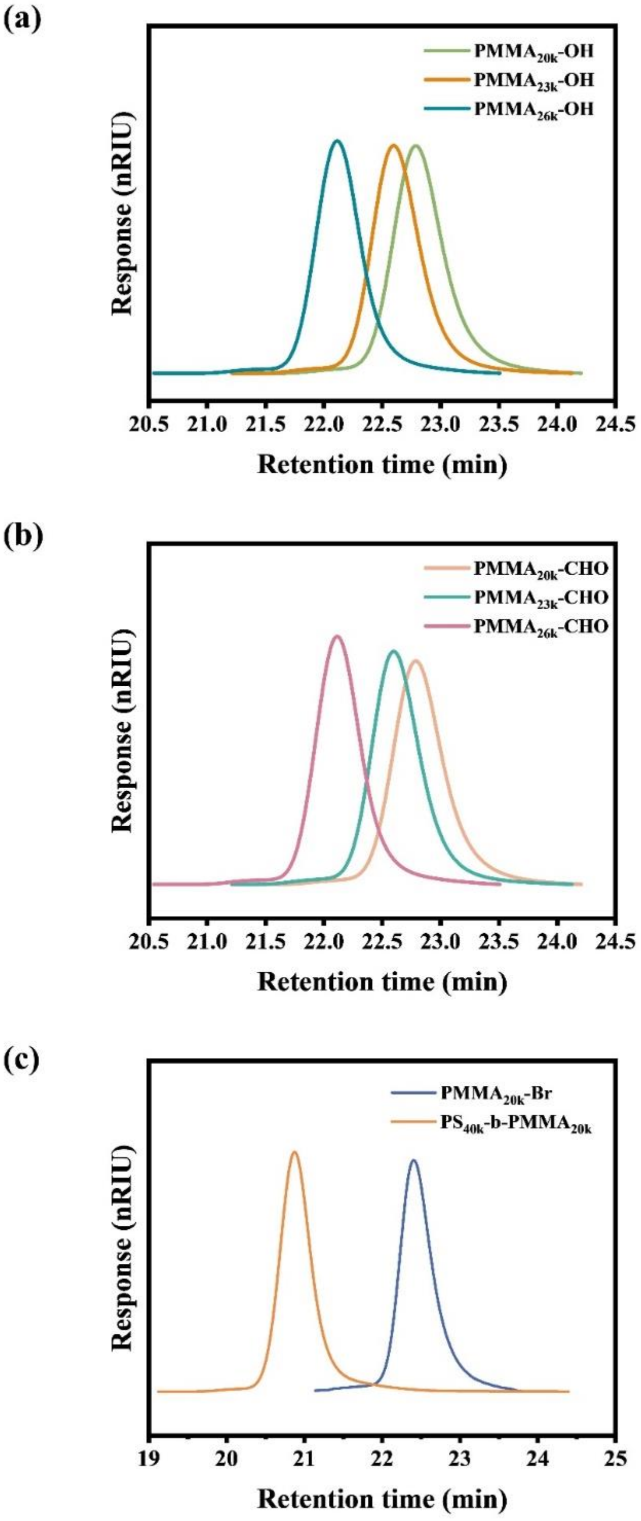

Figure S3. GPC spectra of polymers: (a) PMMA-OH homopolymers, (b) PMMA-CHO homopolymers, and (c) homopolymer $PMMA_{20k}$-Br and block copolymer PS_{40k}-*b*-$PMMA_{20k}$.

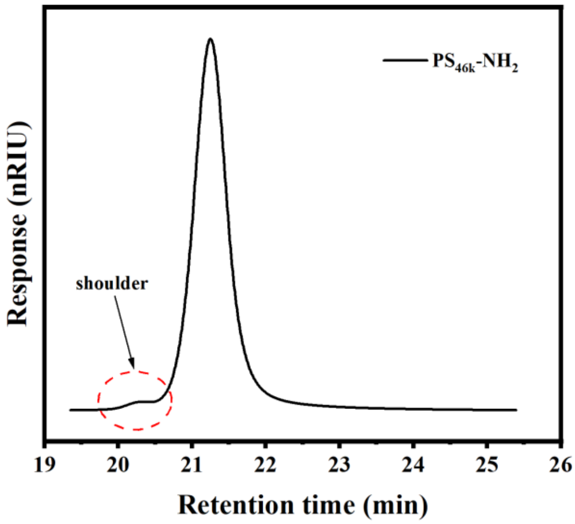

Figure S4. GPC spectrum of commercial amino-terminated polystyrene (PS46k-NH2).

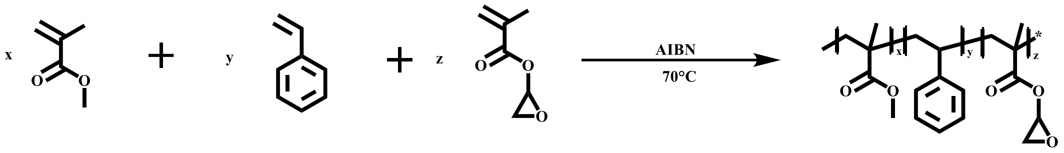

Figure S5. Synthesis scheme of random copolymer mats.

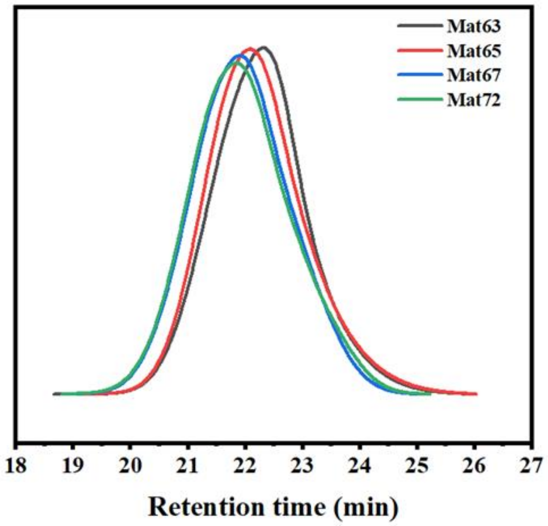

Figure S6. GPC spectra of the random copolymer mats.

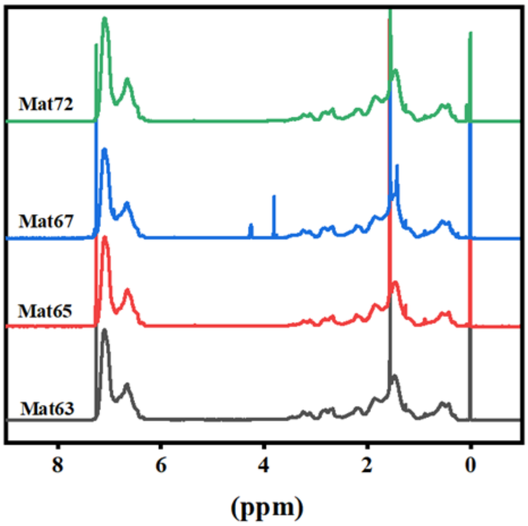

Figure S7. ^{1}H NMR spectra of the random copolymer mats.

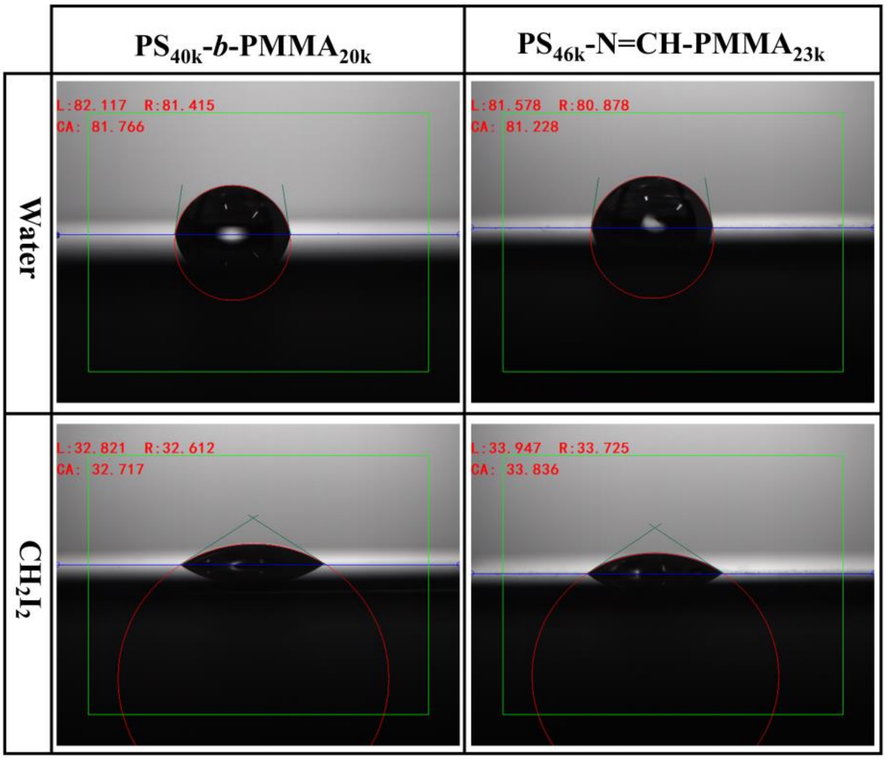

Figure S8. Contact angle measurements for PS_{46k}-N=CH-$PMMA_{23k}$ and PS_{40k}-*b*-$PMMA_{20k}$ were performed using water and diiodomethane.

The surface energy of PS_{46k}-N=CH-$PMMA_{23k}$ and PS_{40k}-b-$PMMA_{20k}$ was measured using the Fowkes equation[1]. The contact angles of water and diiodomethane (CH_2I_2) for each block copolymer film were measured at room temperature using the Fowkes equation.

$$\gamma_L(1+\cos\theta) = 2(\sqrt{\gamma_S^d\gamma_S^d} + \sqrt{\gamma_L^p\gamma_S^p}) \quad \text{(S1)}$$

Table S1. Contact angle measurement and surface energy calculation of PS_{46k}-N=CH-$PMMA_{23k}$ and PS_{40k}-b-$PMMA_{20k}$.

	Contact angle H_2O(°)	Contact angle CH_2I_2(°)	γ (mN m^{-1})
PS_{40k}-b-$PMMA_{20k}$	81.8	32.7	44.1
PS_{46k}-N=CH-$PMMA_{23k}$	81.2	33.8	43.8

The surface energies of PS_{40k}-b-$PMMA_{20k}$ and PS_{46k}-N=CH-$PMMA_{23k}$ are 44.1 mN m^{-1} and 43.8 mN m^{-1}, respectively.

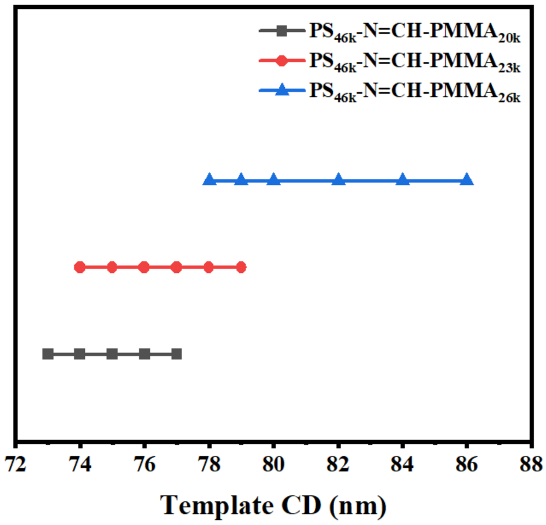

Figure S9. Process window analysis of different PS-N=CH-PMMA samples across the template critical dimension (CD) range.

Table S2. Summary of wetting behaviors of bottom TEOS and sidewall SOC surfaces.

	Without brush	Brush P20255a
SOC sidewall	PMMA-preferential	PS-preferential
TEOS bottom	PMMA-preferential	PMMA-preferential

References

[1] X. Li, J. Li, C. Wang, Y. Liu, H. Deng, *Journal of Materials Chemistry C* **2019**, 7 (9), 2535.
